# Functionalizing Designer DNA Crystals with a Triple-Helical Veneer**

**DOI:** 10.1002/anie.201309914

**Published:** 2014-03-11

**Authors:** David A Rusling, Arun Richard Chandrasekaran, Yoel P Ohayon, Tom Brown, Keith R Fox, Ruojie Sha, Chengde Mao, Nadrian C Seeman

**Affiliations:** Centre for Biological Sciences, University of SouthamptonSouthampton, SO17 1BJ (UK); Department of Chemistry, University of OxfordOxford, OX1 3QZ (UK); Department of Chemistry, Purdue University, West LafayetteIN 47907 (USA); Department of Chemistry, New York UniversityNew York, NY 10003 (USA)

**Keywords:** DNA crystal, nanostructure, self-assembly, tensegrity, triple-helix

## Abstract

DNA is a very useful molecule for the programmed self-assembly of 2D and 3D nanoscale objects.[1] The design of these structures exploits Watson–Crick hybridization and strand exchange to stitch linear duplexes into finite assemblies.[2–4] The dimensions of these complexes can be increased by over five orders of magnitude through self-assembly of cohesive single-stranded segments (sticky ends).[5,6] Methods that exploit the sequence addressability of DNA nanostructures will enable the programmable positioning of components in 2D and 3D space, offering applications such as the organization of nanoelectronics,[7] the direction of biological cascades,[8] and the structure determination of periodically positioned molecules by X-ray diffraction.[9] To this end we present a macroscopic 3D crystal based on the 3-fold rotationally symmetric tensegrity triangle[3,6] that can be functionalized by a triplex-forming oligonucleotide on each of its helical edges.

The tensegrity triangle is a robust motif consisting of three helices directed along linearly independent vectors.[3] By tailing the helices with sticky ends, each triangle can associate with six others, along three different directions, yielding rhombohedral DNA crystals.[6] The triangle is assembled from seven strands; three that partake in the crossovers at the corners, three that extend for the length of each helix, and a final nicked strand completing the crossovers and inner helices at the center. By using seven unique strands, or three strands assembled in a 3:3:1 ratio, triangles have been generated with and without 3-fold rotational symmetry.[6] Crystals have been assembled from tiles containing from two to four helical turns per edge with cavities that have exceeded 1000 nm^3^. It therefore seems plausible that such crystals will be able to host a variety of components, ranging from small molecules, to nanoclusters, to large macromolecules such as proteins.

One approach to introduce extra components into these crystals is by their chemical attachment to the underlying DNA.[10] However, many components, such as proteins, will not tolerate the high temperatures and slow annealing steps required for tile assembly and side interactions might also influence, or disrupt, the assembly pathway. A more robust strategy is to address the duplex sequences within the tile using a DNA recognition agent.[11,12] Coupling a component to such an agent will lead to its targeted introduction at these sites. To this end, we have demonstrated that the triplex approach to DNA recognition[13] can be exploited to incorporate guest molecules at unique locations within a tensegrity triangle crystal (Figure 1). Triplex-forming oligonucleotides (TFOs) are sequence-specific recognition agents that bind within the major groove of duplex DNA by generating base triplets; pyrimidine-containing TFOs bind in a parallel orientation to the purine strand of the target duplex, generating C^+^⋅GC and T⋅AT triplets (Figure 1 A). An appropriate 13 base pair target site (Figure 1 B) was therefore embedded within a previously reported 3-fold symmetric tile that contains three turns per helix (3TS; Figure S1 in the Supporting Information).[6] This generated a modified tile containing three sites capable of being targeted by a TFO (3TS-mod; Figure 1 D). Since triplex formation usually requires conditions of low pH (<6.0) we also prepared TFOs that contain the triplex-stabilizing nucleosides 2′-aminoethoxy-methyl-C and 2′-aminoethoxy-T in place of C and T, respectively (Figure 1 C).[14] To aid in characterization, and to demonstrate the feasibility of introducing other non-nucleic acid molecules, each TFO contained a cyanine dye (Cy5) attached through a C6-linker to its 5′-terminus.

**Figure 1:**
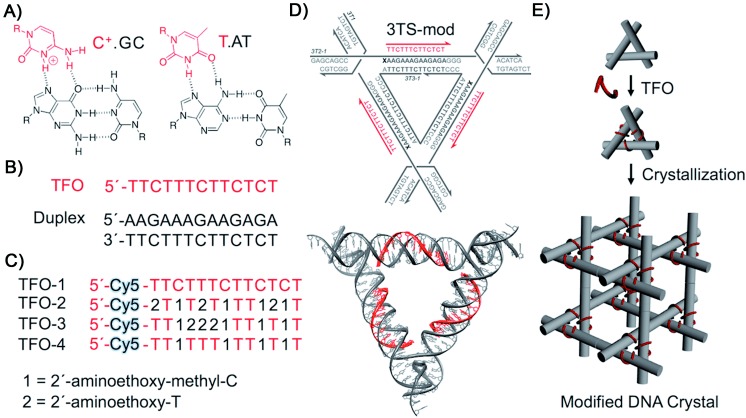
Targeting the tensegrity triangle. A) Base triplets. B) Triplex sequence. C) Cy5-labeled TFOs containing stabilizing analogues. D) Sequence and model of the TFO-bound tile (see Figure S2). Tiles were generated with T or FAM-C6-dT at position X (in bold) with the latter referred to as F-3TS-mod. E) Strategy for the functionalization of DNA crystals.

The objective of this study was to bind the TFO to the pre-assembled tile at a stage prior to the crystallization step (Figure 1 E). We determined whether each TFO bound to the tiles by an electrophoretic mobility shift assay (EMSA) (Figure 2). 3TS and 3TS-mod tiles were annealed and incubated with each TFO in TA-Mg buffer at pH 5 or 7. Both tiles without TFO reveal single bands with identical mobility, confirming that the sequence difference of the 3TS-mod tile has not influenced its assembly (lanes 1 and 6). As expected, the TFOs did not affect the mobility of the unmodified 3TS tile at both pH (lanes 2–5), but produce bands with slower mobility with the 3TS-mod tile. At pH 5 all four TFOs reduce the mobility, while at pH 7 only TFO-2 and TFO-3 resulted in a full shift; the unmodified TFO-1 shows no interaction, while TFO-4 only affects about 50 % of the tile (lanes 7–10). Experiments examining the concentration dependence of the interaction showed the greatest shift with a 1:1 ratio of TFO:target site, suggesting full site occupancy (Figure S3). The TFOs can also be introduced before triangle assembly by using much faster annealing rates, allowing the duplex regions of the tile to assemble before the slower binding of the TFO (Figure S4 and S5).

**Figure 2:**
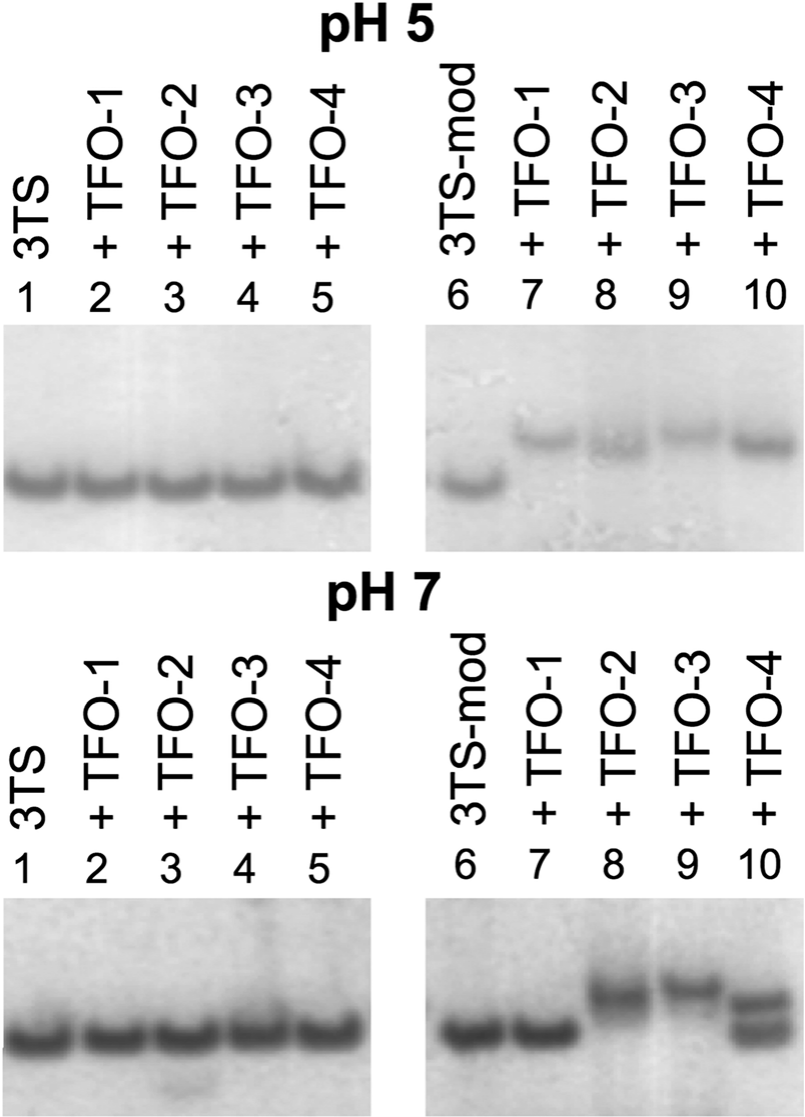
TFO–tile EMSA. Each tile was annealed at a concentration of 4 μm before addition of TFOs 1–4 at a concentration of 12 μm (i.e., 1:1 ratio of TFO:sites). Samples were run on a non-denaturing 8 % polyacrylamide gel in TA-Mg buffer at 4 °C and subjected to post-staining.

The location of the bound TFO was established by subjecting the modified tiles to an enzymatic protection assay. The non-crossover strand of the tile was labeled with ^32^P at its 5′ end, annealed with the remaining tile oligonucleotides, and incubated with each TFO at pH 7. Samples were subsequently digested by DNase I under “limiting conditions” and the cleavage products separated by denaturing polyacrylamide gel electrophoresis (Figure 3 and S6). A double-stranded equivalent, containing the radiolabeled DNA with its fully complementary strand, was included for comparison. In the absence of TFO the cleavage patterns for the tile and duplex show some marked differences as the crossover points occlude cleavage by the enzyme (lanes 1 and 3; black arrows). By comparing these lanes with those in the presence of the TFOs additional bands are missing (footprint) only at the location of the oligopurine target site, indicating that TFO binding has prevented digestion by the enzyme (Figure 3; lanes 2, 4–6; light gray arrows). No footprints were seen for TFO-1 with either the tile or its duplex equivalent, while TFO-4 only generated a footprint with the duplex equivalent (Figure S6). However, both TFOs were capable of interacting with their intended duplex sites at pH 5 (Figure S7). A characteristic of DNase I footprints generated by TFOs is hypersensitivity at the triplex–duplex junction at the 3′ end of the oligopurine strand and this is again observed here (dark gray arrow).[12]

**Figure 3:**
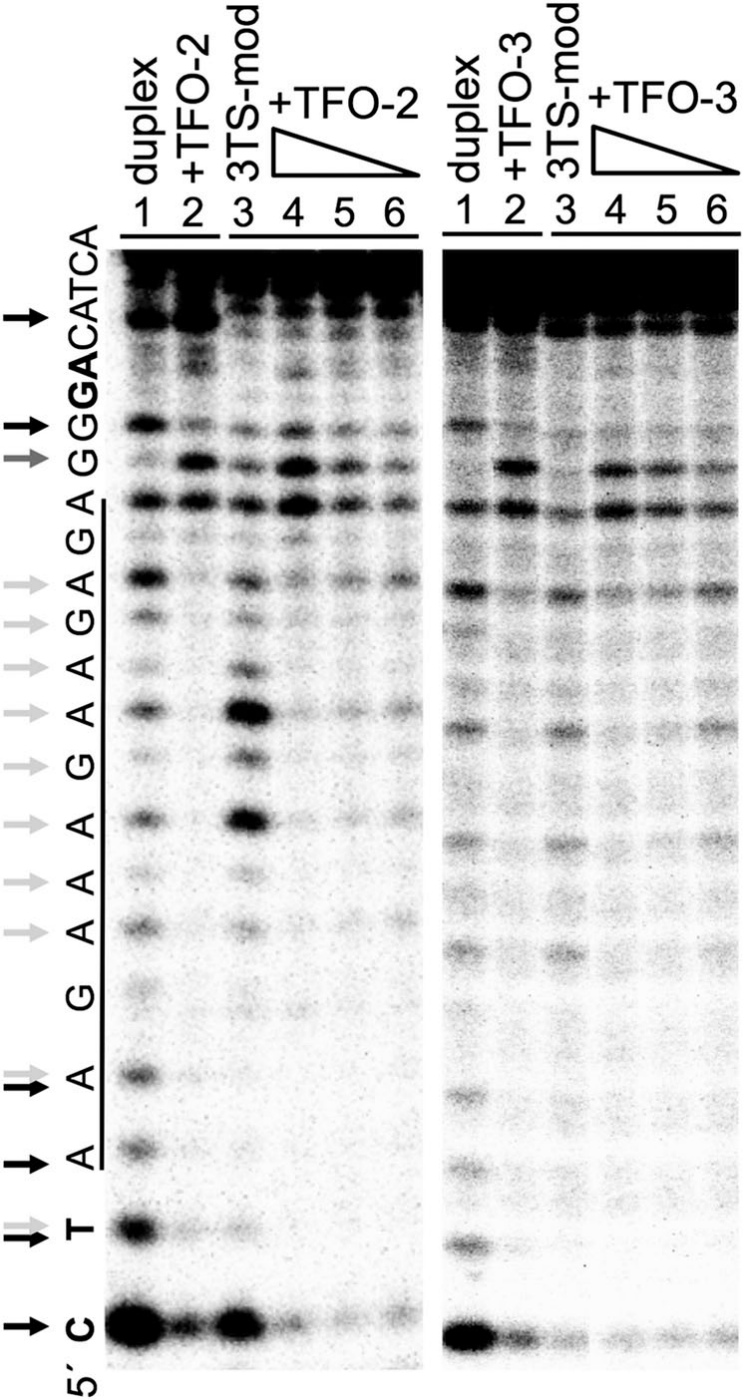
TFO–tile protection assay. The non-crossover strand was labeled with ^32^P at its 5′ end and annealed at a final concentration of 100 nm. The tile was digested by DNase I before or after incubating with the TFOs at a concentration of 1, 0.3 and 0.1 μm at 4 °C. Samples were run on a denaturing 22 % polyacrylamide gel and subjected to phosphor-imaging. A duplex control was digested in the presence and absence of 1 μm of TFO for comparison. The sequence of the labeled strand is shown on the left of the gels, underlined regions represents the TFO binding site and bold letters the bases flanking each side of the crossover points (triangle only). Black arrows highlight bands missing between the triangle and duplex control; light gray arrows bands missing due to binding of the TFO and the dark gray arrow a band showing DNase I hypersensitivity at the triplex–duplex junction.

To obtain a quantitative estimate of the stability of each of the triplexes, the complexes were examined with thermal denaturation experiments. Fluorescein was attached to a T located adjacent to the 5′ end of the oligopurine target site within the tile (F-3TS-mod; Figure 1 D) and annealed with the remaining unmodified strands. Upon triplex formation with a Cy5-labeled TFO, the two dyes are in close proximity and capable of fluorescence resonance energy transfer (FRET). The fluorescein was excited at 488 nm and emission of the Cy5 label was measured at 710 nm. Upon heating the TFO dissociated from the tiles resulting in a decrease in fluorescence, observed as a melting curve (Figure 4). Melting temperatures (*T*_m_) were then calculated from the midpoints and revealed the order of thermal stability of the triplexes to be TFO-3 > TFO-2 > TFO-4 > TFO-1 (Table S1). TFO-3, which contains nucleoside modifications positioned adjacent to one another, produced the most stable triplex, in agreement with a previously reported study.[15] The experiment was repeated using an equivalent duplex that contained a strand that was fully complementary to the labeled oligopurine-containing strand; nevertheless, the order of stabilities remained the same (Figure S8). To confirm the fluorescein did not influence assembly melting curves were determined by UV melting using unmodified tiles (Figure S9). The calculated melting temperatures (*T*_m_) were all within 1 °C of those determined by FRET melting (Table S1).

**Figure 4:**
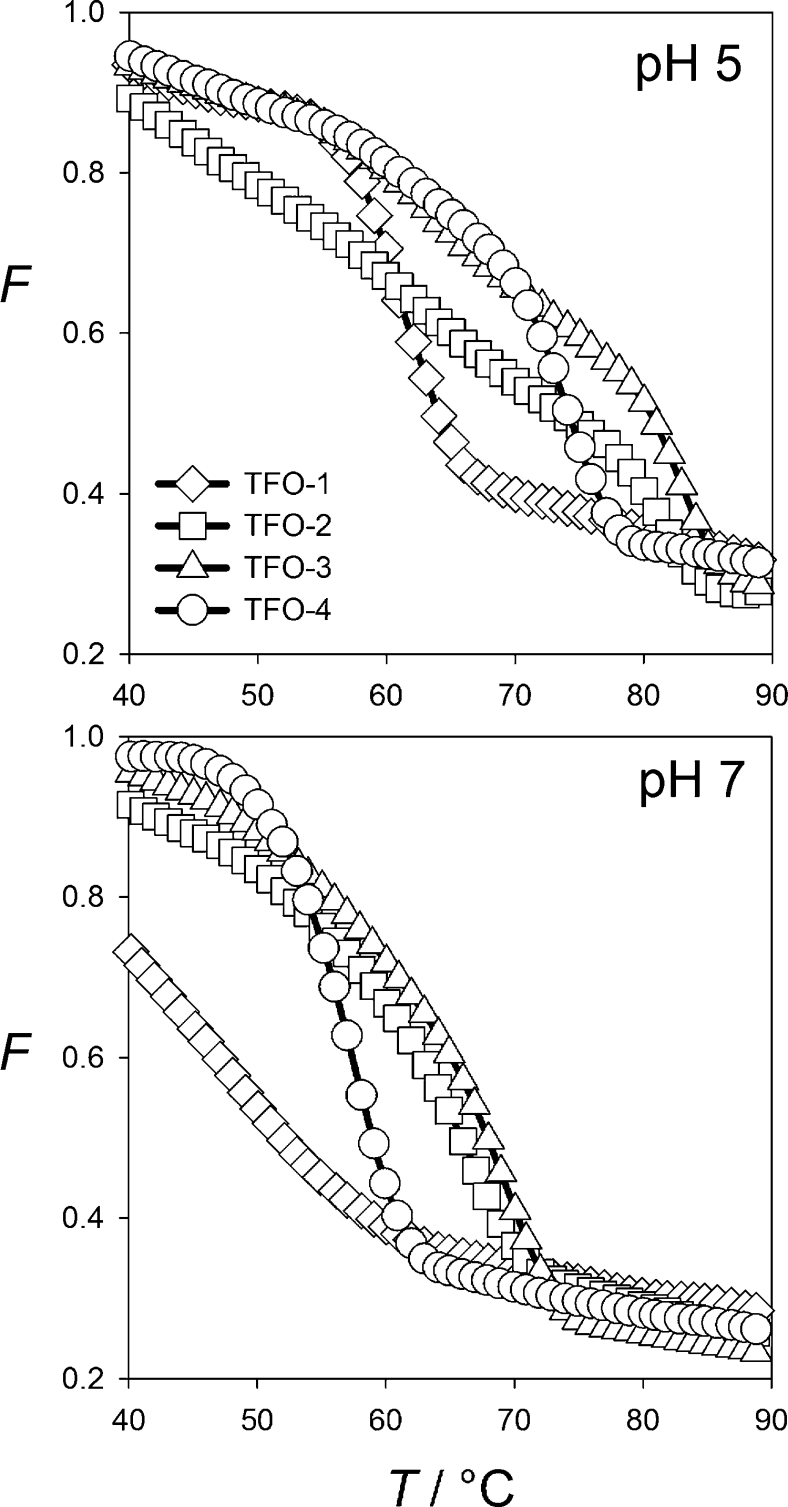
TFO–tile melting curves. The non-crossover strand of the tile was labeled with FAM-C6-dT at a position adjacent to the triplex target site, while TFOs 1–4 contained an attached Cy5 dye. Before melting the labeled strand was annealed at a final concentration of 0.1 μm before addition of TFOs 1–4 at a concentration of 1000 nm. Melting profiles were recorded at a rate of 0.2 °C min^−1^. Fluorescence emission was recorded from the Cy5 dye at 710 nm after excitation of the fluorescein at 488 nm.

We next investigated whether the TFO-modified tiles were capable of self-assembled crystal growth. Each of the modified tiles was assembled as before and crystals grown from hanging drops containing TA-Mg buffer equilibrated against a reservoir of ammonium sulfate. Crystals obtained at pH 5 in the presence of TFO-1 are shown in Figure 5. Crystallization of the unmodified 3TS tile with TFO-1 did not affect crystal formation and generated rhombohedral crystals similar in morphology to those previously reported (Figure 5 A).[6,10] The acidic pH also did not disrupt crystallization. Incubation of TFO-1 with the 3TS-mod tile yielded turquoise colored crystals, clearly indicating incorporation of the cyanine dye within the crystal (Figure 5 B). The bound TFO has therefore not disrupted the self-assembly of the individual tiles into crystals. This experiment was repeated with each TFO at both pH 5 and pH 7 and similar results were obtained (Figure S10). The formation of the crystals at pH 7 with TFO-1 was surprising as no complex formation was detected in the above experiments, though crystallization conditions may favor TFO binding to the tile as a result of the greater oligonucleotide concentrations. A similar experiment examined the formation of crystals with the fluorescein-labeled tiles (F-3TS-mod) in the presence and absence of TFO-1 (Figure 5 C and D). In the absence of TFO the crystals were yellow, whilst in the presence of TFO the crystals were green, again demonstrating successful incorporation of the TFO within the crystal. Several of the TFO-modified crystals were also subjected to X-ray diffraction analysis, yielding diffraction patterns comparable in resolution to those previously obtained for the 3TS crystal in the absence of TFO (ca. 6 Å).[6]

**Figure 5:**
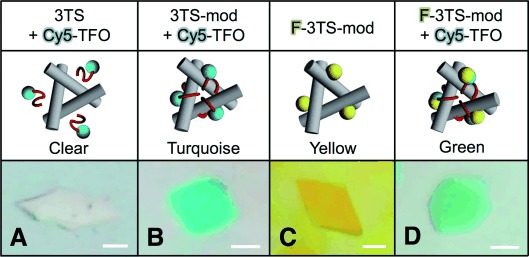
TFO-modified crystals. TFO-bound tiles were assembled as before and crystals grown at 4 °C. Concentrations of the tiles and TFO-1 before crystallization were 4 and 12 μm, respectively. Representative crystal pictures were taken in the absence of polarizer and the scale bar represents 50 μm.

DNA recognition is an attractive method for introducing functionality into complex DNA architectures since it overcomes problems associated with prior attachment of a component to the underlying DNA scaffold. TFOs offer the advantage that they are compatibility with a wide variety of oligonucleotide conjugation strategies. In addition, the pH dependence and target site requirements can be overcome using stabilizing nucleoside modifications.[16] The targeting of a single component to each helix within the 3TS crystal dictates its positioning with sub-nanometer precision; each component is separated by ca. 10.5 nm along the helix axis between tiles and 5.8 nm through 3D space within the same tile (i.e., between the 5′ ends of each TFO). Since a typical 100 μm crystal contains an estimated 10^12^ unit cells, full occupancy of each binding site within the crystal would result in the incorporation of the same number of periodically repeating components within the crystal. The housing of three components within the crystal’s 366 nm^3^ rhombohedral cavities increases the local concentration of the components drastically (ca. 14 mm). This could be exploited for example, to enhance, or direct, enzymatic cascades.[8] In addition, the incorporation of a single guest molecule within the asymmetric unit cell of the crystal would be optimal for their X-ray crystallographic analysis. Owing to the three-fold rotational symmetry of the 3TS crystals this will require full occupancy of each of the three binding sites per triangle; three-fold averaging of a single guest would not be optimal for determining structures. Although it is not possible to determine the occupancy of each binding site within these symmetric crystals, biophysical experiments with the tiles before crystallization suggest full occupancy, and this is unlikely to be influenced by self-assembled crystal growth. An alternative strategy is to target a binding site located on a single helix within an asymmetric tile system, removing the requirement for 3-fold averaging.[6] The diffraction resolution of these crystals is also relatively low (6 Å) but this could be improved by targeting tiles designed to contain two (not three) helical turns per edge that have been shown to diffract to a higher resolution (ca. 4 Å).[6] Exploiting such designer crystals as macromolecular hosts could therefore help alleviate the macromolecular crystallization problem, and we continue to work towards this goal.
