# Supplementary material for: Functionalizing Designer DNA Crystals with a Triple-Helical Veneer
Source: Angew Chem Int Ed Engl. 2014 Mar 11;53(15):3979–82. doi: 10.1002/anie.201309914 (PMC4037404; doi:10.1002/anie.201309914)
Supplement: Supplementary file 1 [file anie0053-3979-sd1.pdf]

Angewandte Chemie
Eine Zeitschrift der Gesellschaft Deutscher Chemiker

Supporting Information
© Wiley-VCH 2014
69451 Weinheim, Germany

Functionalizing Designer DNA Crystals with a Triple-Helical Veneer**

David A. Rusling, Arun Richard Chandrasekaran, Yoel P. Ohayon, Tom Brown, Keith R. Fox, Ruojie Sha, Chengde Mao, and Nadrian C. Seeman**

anie_201309914_sm_miscellaneous_information.pdf

Experimental Procedures

Oligonucleotides and synthesis Oligonucleotides were synthesized on an Applied Biosystems ABI 394 automated DNA/RNA synthesizer on the 0.2 µmole scale using standard procedures or purchased from Integrated DNA Technologies. Phosphoramidite monomers and other reagents were obtained from Applied Biosystems, Link Technologies and Glen Research. The phosphoramidite monomer for the 2´-aminoethoxy-T nucleoside was synthesized as previously described,[1] while the synthesis of the phosphoramidite monomer for the 2´-aminoethoxy-5-methyl-C nucleoside will be reported elsewhere. Oligonucleotides were unmodified except for TFOs 1-4, which contained a Cy5 dye attached *via* a C6 linker to the 5´-terminal phosphate and various internal substitutions of 2´-aminoethoxy-5-methyl-C and 2´-aminoethoxy-T nucleosides, and F-3T2-1, which contained a fluorescein dye attached *via* a C6 linker to an internal dT residue. The full sequences of the oligonucleotides used in this study are shown on page 3.

Tile assembly Oligonucleotides for each tile were mixed stoichiometrically in pH 5.0 or pH 7.0 TA-Mg buffer (40 mM tris-acetate containing 15 mM magnesium acetate) and annealed using the following protocol: 90 °C for 5 minutes, 65 °C for 20 minutes, 45 °C for 20 minutes, 37 °C for 30 minutes and 20 °C for 30 minutes. Unless otherwise stated the TFOs were added after tile assembly and left to equilibrate at 4 °C for > 8 hrs.

Electrophoretic mobility shift assay (EMSA) TFO binding by the 3TS and 3TS-mod tiles was examined by subjecting the complexes to an EMSA. The final concentration of the tiles and the TFOs was 4 and 12 µM, respectively. Complexes were run on a non-denaturing 8% polyacrylamide gel in the appropriate TA-Mg buffer at 4 °C and visualized by subjecting the gel to post-staining with Stains-All (Sigma Aldrich).

Enzymatic protection assay The location of the bound TFOs within the 3TS-mod tile was determined by DNase I footprinting.[2] The non-crossover oligonucleotide of the tile (3T2-1) was first phosphorylated at its 5´-end with γ-^{32}P[ATP] using T4 polynucleotide kinase (New England Biolabs) and purified by denaturing PAGE. It was then annealed with the remaining tile (or duplex) oligonucleotides at an

estimated final concentration of 0.1 μM. Each TFO in turn was incubated with the tile (or duplex) at a final concentration between 1 and 10 μM and left to equilibrate overnight. The resulting complexes were mixed with 2 μl of DNase I (typically 0.01 units/ml) dissolved in 20 mM NaCl containing 2 mM $MgCl_2$ and 2 mM $MnCl_2$. The reaction was stopped after 1 min by adding 4 μl DNase I stop solution [80% formamide, 10 mM EDTA, 10 mM NaOH, and 0.1% (w/v) bromophenol blue]. The products of digestion were separated on a denaturing 22% polyacrylamide gel and the gel subjected to phosphorimaging.

Fluorescence (FRET) melting Thermal melting profiles for the complexes were determined using molecular beacons and a Roche LightCycler.[3] TFOs were labeled at their 5′ -end with Cy5 while the purine-containing strand (F-3T2-1) of the tile (or duplex control) was labeled at a corresponding internal position with fluorescein. Upon complex assembly the two dyes are in close proximity and are capable of fluorescence energy transfer (FRET). The final concentration of the tile and the TFOs was 0.1 and 1 μM, respectively. Melting profiles were obtained by heating the samples to 95 °C at a rate of 0.2 °C min^{-1} and measuring the fluorescence emission from the Cy5 dye at 710 nm after excitation of the fluorescein at 488 nm. [Although the slowest rate of continuous temperature change in the LightCycler is 0.1 °C s^{-1}, slower melting profiles were obtained by increasing the temperature in 1 °C steps, leaving the samples to equilibrate for a set amount of time.] The complexes were then cooled at an identical ramp rate to check for hysteresis and both the melting and annealing temperatures (T_m) varied by less than 1 °C. T_m values were determined from the first derivatives of the melting and annealing profiles using the software provided with the machine and differed by less than 0.5 °C between experiments.

Ultraviolet (UV) melting Thermal melting profiles for the complexes were determined by measuring their UV absorbance at 260 nm with a Cary 100 Bio UV-Visible Spectrophotometer as the samples were heated at a rate of 0.15 °C min^{-1}. The final concentration of the tile and the TFOs was 0.5 and 1.5 μM, respectively. T_m values were determined from the first derivatives of the melting profiles using the software provided with the machine and differed by less than 1 °C between experiments.

Crystallization The TFO bound tiles were assembled as above with the tiles and TFOs at a final concentration of 4 and 12 μM, respectively. Crystals were grown from 5 μl hanging drops containing TA-Mg buffer and equilibrated against a 600 μl reservoir of 1.75 M ammonium sulfate.[4] The whole set-up was kept at 4 °C until crystals were obtained (typically 4-5 days).

<u>Oligonucleotide Sequences</u>

Tiles:

3TS tile

3T1: 5′ -TCTGATGTGGCTGC

3T2: 5′ -GAGCAGCCTGTACTCGGCTTGTCGGACATCA

3T3: 5′ -CCGAGTACACCGACAAGCCGAGTACACCGACAAGCCGAGTACACCGACAAG

3TS-mod tile (with **3T1**)

3T2-1: 5′ -GAGCAGCCTAAGAAAGAAGAGAGGGACATCA

3T3-1: 5′ -TCTTTCTTACCCTCTCTTCTTTCTTACCCTCTCTTCTTTCTTACCCTCTCT

F-3TS-mod tile (with **3T1** and **3T3-1**)

F-3T2-1: 5′ -GAGCAGCC**X**AAGAAAGAAGAGAGGGACATCA (where **X** is FAM-C6-dT)

1TA duplex (with **3T2-1**)

Dup1: 5′ -TGATGTCCCTCTCTTCTTTCTTAGGCTGCTC

***Triplex-forming oligonucleotides* (TFOs):**

TFO-1: 5′ -**Cy5**-TTCTTTCTTCTCT

TFO-2: 5′ -**Cy5**-**2**T**1**T**2**T**1**TT**121**T

TFO-3: 5′ -**Cy5**-TT**12221**TT**1**T**1**T

TFO-4: 5′ -**Cy5**-TT**1**TTT**1**TT**1**T**1**T

(where **1** is 2′-aminoethoxy-5-methyl-C and **2** is 2′-aminoethoxy-T)

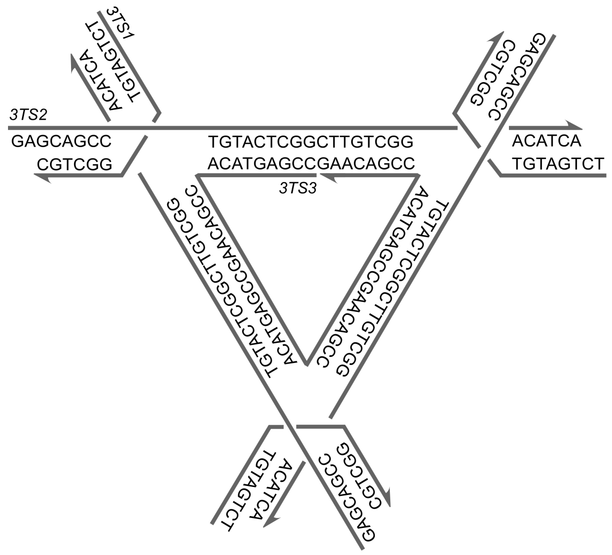

Figure S1: The 3TS tile. Full sequence of the previously reported[5] three-turn symmetric tile (3TS) tile that was modified to include an oligopurine-oligopyrimidine TFO target site at each of its three helical edges (3TS-mod).

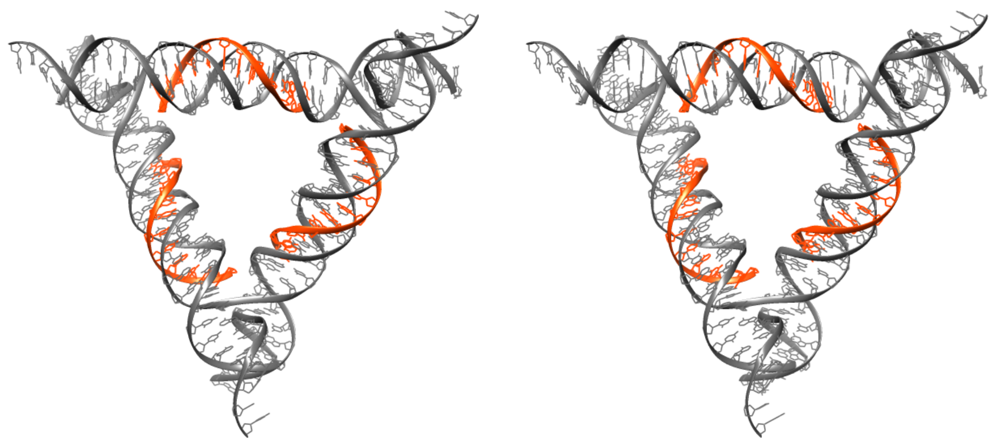

Figure S2: Stereoscopic model for a triplex-modified 3TS-mod tile.

Additional Data

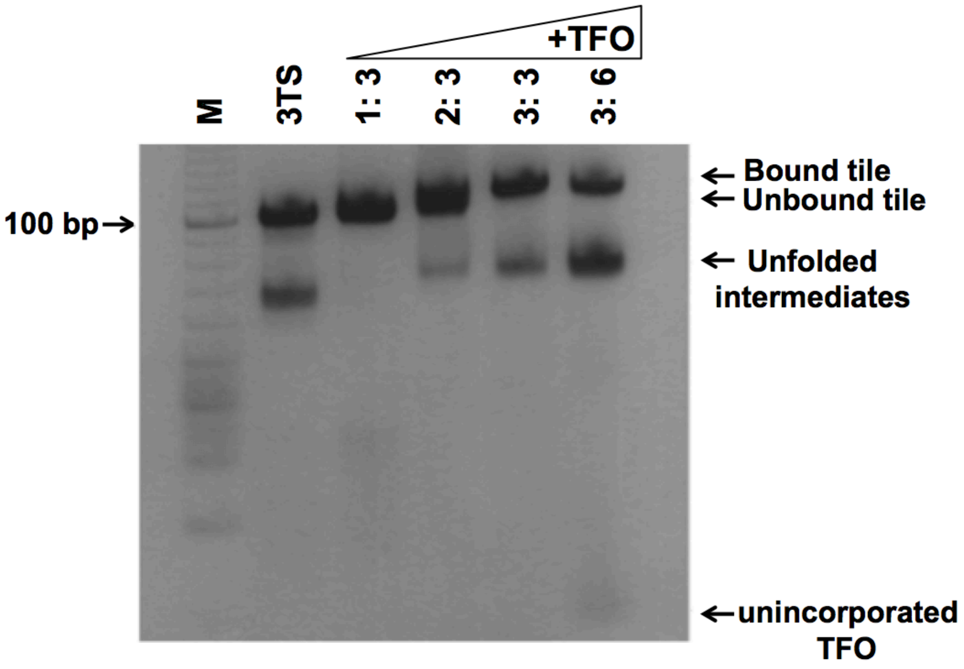

Figure S3: Concentration dependence. The 3TS-mod tile was annealed in pH 5.0 TA-Mg buffer at a final concentration of 4 μM before the addition of TFO-1 at a final concentration of 4, 8, 12 and 24 μM, *i.e.* a molar ratio of 1:3, 2:3, 3:3 and 6:3 of TFO:TFO binding sites, respectively. Samples were run on a non-denaturing 8% polyacrylamide gel in pH 5 TA-Mg buffer at 4 °C and the complexes visualized by subjecting the gel to post-staining with Stains-All (Sigma Aldrich). The mobility of the tile is influenced by the number of occupied binding sites; as the TFO concentration increases the mobility of the tile decreases until each of the binding sites becomes occupied by the TFO (**lane 3-6**).

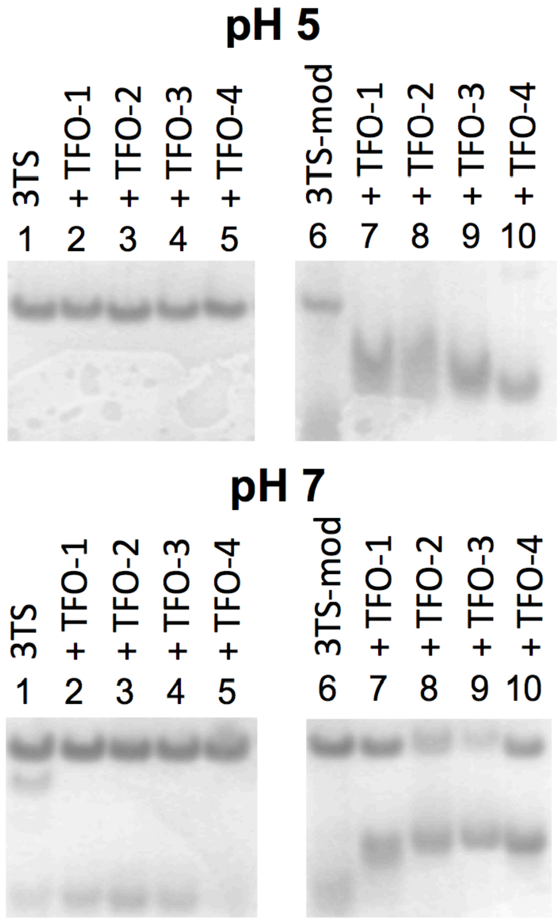

Figure S4: Tile assembly is influenced by TFO addition. The 3TS and 3TS-mod tiles were annealed in TA-Mg buffer at a final concentration of 4 μM in the presence of TFOs 1-4 at a final concentration of 12 μM (*i.e.,* a molar ratio of 1:1 of TFO:number of TFO binding sites). Samples were run on a non-denaturing 8% polyacrylamide gel in TA-Mg buffer at 4 °C and the complexes visualized by subjecting the gel to post-staining with Stains-All (Sigma Aldrich). Assembly of the 3TS tile is not influenced by the presence of the TFO. In contrast, assembly of the 3TS-mod tile is disrupted by the presence of the TFO and leads to unfolded intermediates; this is most evident at pH 5 when the TFOs have the highest affinity for their target sites within the tile (**lower bands; lanes 7-10**).

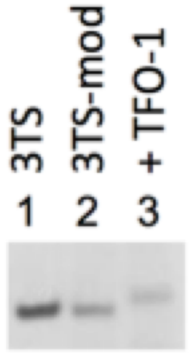

Figure S5: Tile assembly at faster rates of annealing (10.5 °C min^{-1}). The 3TS-mod tile was annealed in pH 5.0 TA-Mg buffer at a final concentration of 4 μM in the presence of TFOs 1-4 at a final concentration of 12 μM (*i.e.*, a molar ratio of 1:1 of TFO:number of TFO binding sites). Samples were run on a non-denaturing 8% polyacrylamide gel in pH 5 TA-Mg buffer at 4 °C and the complexes visualized by subjecting the gel to post-staining with Stains-All (Sigma Aldrich). A shifted band that corresponds to the fully assembled TFO bound-tile is observed at the faster rate of annealing (**lane 3**).

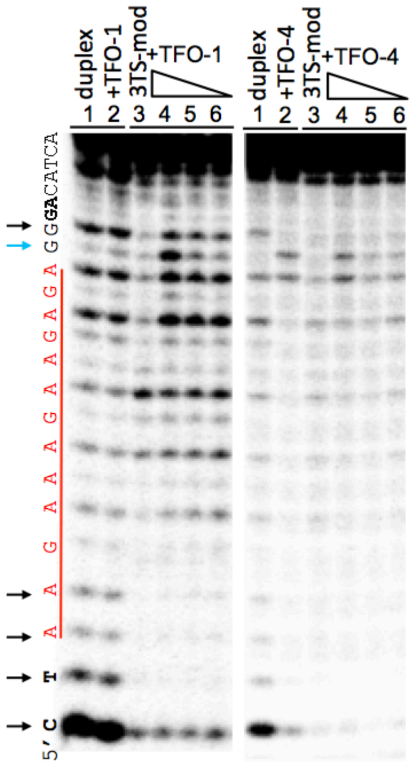

Figure S6: Protection from DNase I cleavage reveals TFO-1 and 4 lack affinity for the tile at pH 7. The non-crossover strand of the tile was labelled with ^{32}P at its 5′ -end and subsequently annealed with the remaining strands to a final concentration of 100 nM. The tile was then digested with DNase I either before (lane 3) or after incubating the tile with TFO at a final concentration of 1000, 300 and 100 nM for > 8h at 4 ℃ (**lane 4-6**). Samples were run on a denaturing 22% polyacrylamide gel and subjected to phosphorimaging. An additional duplex control containing the labelled non-crossover strand and its complementary strand (dup-1) was also digested in the presence and absence of 1000 nM of TFO (**lanes 1 and 2**). The sequence of the labelled non-crossover strand is shown on the left of the gel, underlined sequences in red represent the intended TFO binding site and the bold letters reflect the bases flanking each side of the crossover points (triangle only). Black arrows highlight bands that are missing in the cleavage pattern of the triangle relative to the duplex control and reflect regions that are inaccessible to DNase I cleavage, such as at the crossover points (**lanes 1 and 3**). Although no clear footprints are seen for either of the TFOs the blue arrow indicates a band that is enhanced due to DNase I hypersensitivity at the triplex-duplex junction, suggesting a weak interaction.

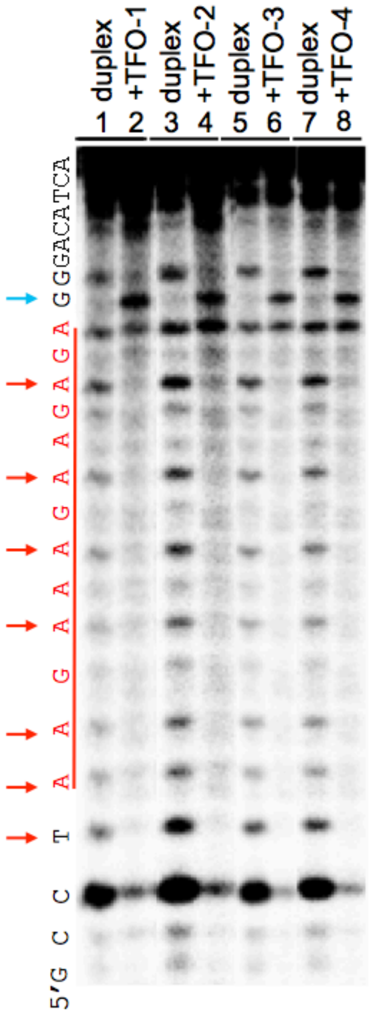

Figure S7: Protection from DNase I cleavage reveals duplex binding by TFOs 1-4 at pH 5. The non-crossover strand of the tile was labelled with ^{32}P at its 5′ -end and annealed with a complementary duplex strand (Dup-1) to a final concentration of 100 nM. The duplex was then digested with DNase I either before or after incubating the tile with TFO at a final concentration of 1000 nM for > 8h at 4 °C. Samples were run on a denaturing 22% polyacrylamide gel and subjected to phosphorimaging. The sequence of the labelled non-crossover strand is shown on the left of the gel; underlined sequences in red represent the intended TFO binding site. Red arrows highlight bands that are missing in the cleavage pattern due to TFO binding and the blue arrow a band that is enhanced due to DNase I hypersensitivity at the triplex-duplex junction. As expected, all TFOs interact with the duplex at pH 5.0.

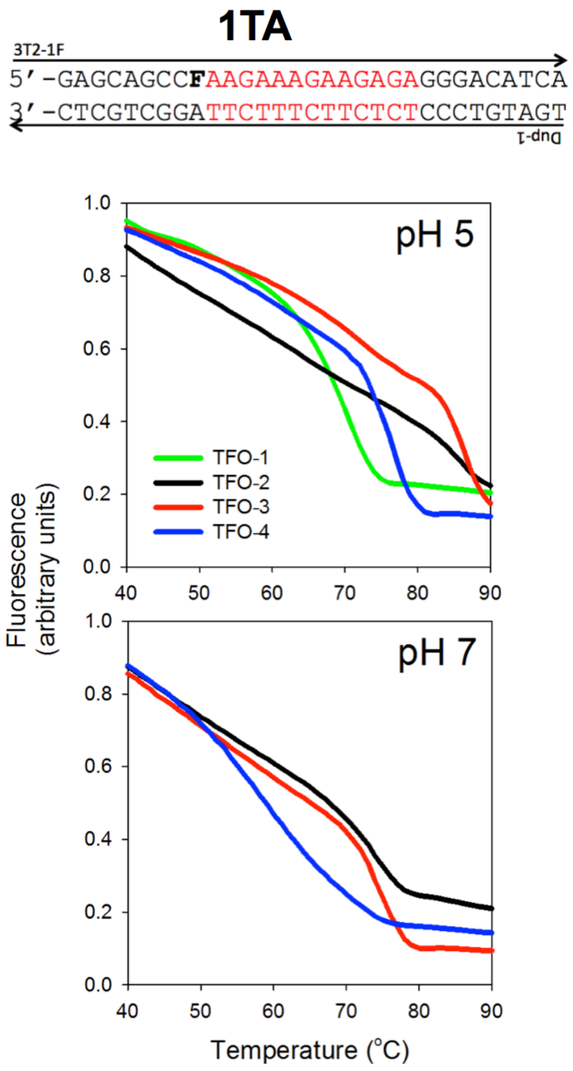

Figure S8: Fluorescence melting of the 1TA duplex in the presence of TFOs 1-4 at pH 5 and 7. The top strand of the duplex was labelled internally with FAM-C6-dT (F) at a position adjacent to the triplex target site, while TFOs 1-4 contained an attached Cy5-dye. Before melting the labelled strand was annealed with its complementary duplex strand (Dup-1; bottom) at a final concentration of 100 nM before addition of TFO 1-4 at a final concentration of 1000 nM. Fluorescence melting profiles were recorded in a Roche LightCycler by heating the samples at a rate of 0.2 °C min^{-1}. Fluorescence emission was recorded from the Cy5 dye at 710 nm after excitation of the fluorescein at 488 nm. The order of thermal stability of the triplexes generated by TFOs 1-4 is TFO-3 > TFO-2 > TFO-4 >> TFO-1 at both pH values.

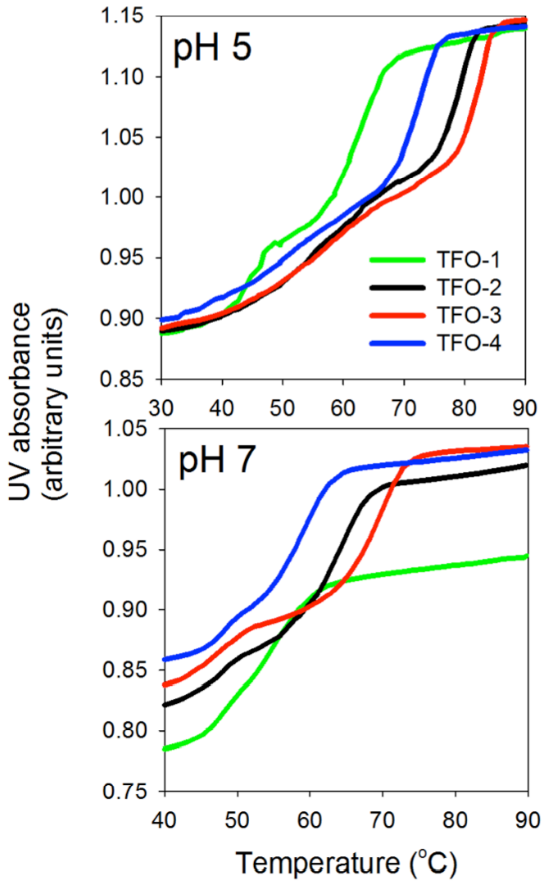

Figure S9: Melting profiles obtained from UV melting of the 3TS tile in the presence of TFOs 1-4. The 3TS-mod tile was annealed in TA-Mg buffer at a final concentration of 0.5 μM in the presence of TFOs 1-4 at a final concentration of 1.5 μM, *i.e.,* a molar ratio of 1:1 of TFO:number of TFO binding sites. The complexes were then annealed at 4 °C and left to equilibrate for > 8 hrs. Melting profiles were recorded using a Cary 100 Bio UV-Visible Spectrophotometer by heating the samples at a rate of 0.15 °C/min and measuring the change in absorbance at 260 nm. In each case two melting transitions are evident; the first is attributed to the dissociation of the smaller duplex regions at each of the triangle corners, while the second is due to the dissociation of the more stable triplex regions at the triangle centre.

Table S1: Comparison of melting temperatures (T_ms) calculated from thermal denaturation experiments. In most cases values differed by less than a single degree.

	**Melting temperatures (T_ms)**			
	**pH 5.0**		**pH 7.0**	
	**FRET**	**UV**	**FRET**	**UV**
**TFO-1**	61	62	-	-
**TFO-2**	81	80	65	65
**TFO-3**	82	83	69	70
**TFO-4**	74	73	57	59

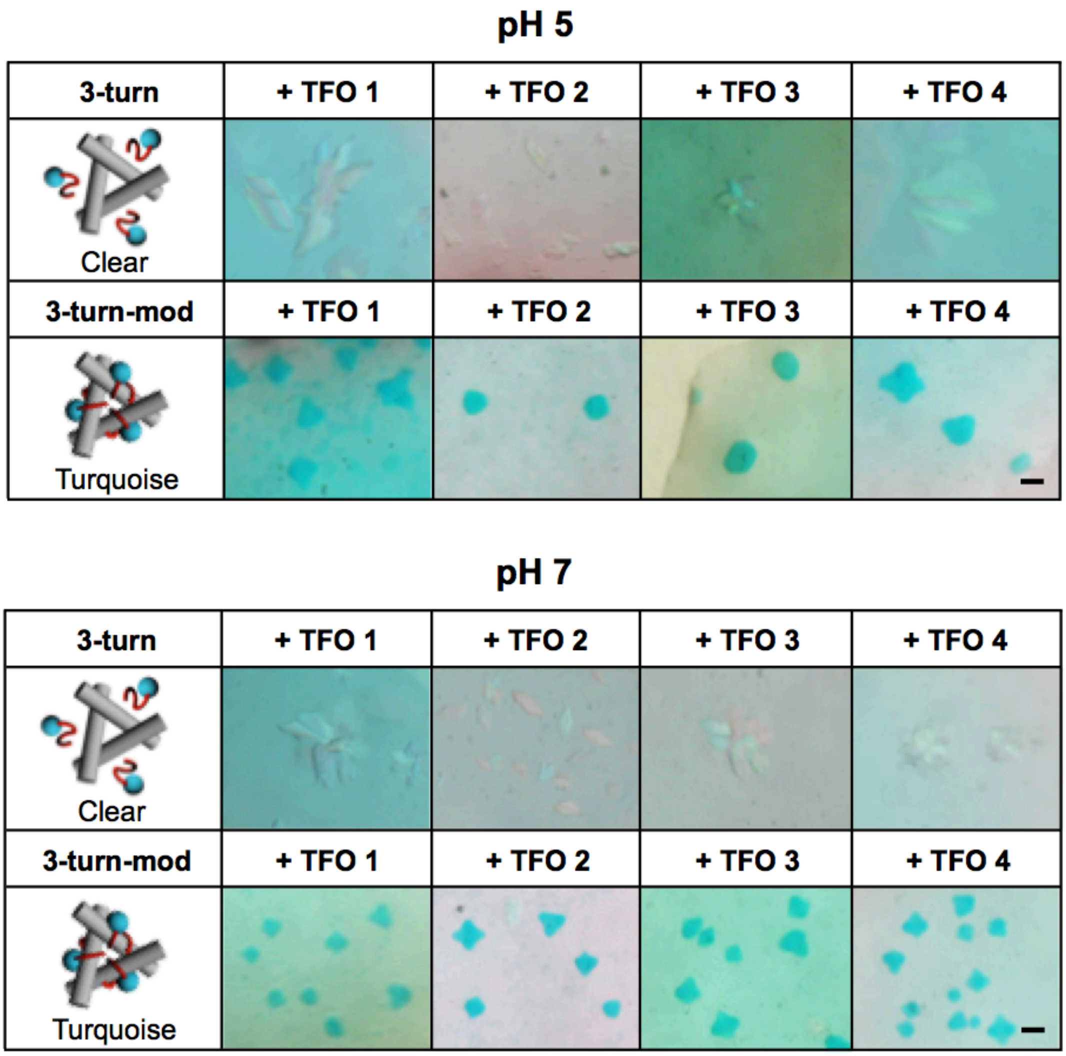

Figure S10. Crystal color change demonstrates the incorporation of TFO 1-4 within the 3TS-mod crystal. Crystals were grown in sitting drops and equilibrated against a reservoir of ammonium sulfate. The final concentrations of the tiles and TFOs were 4 and 12 μm, respectively. The representative crystal pictures were taken in the absence of polarizer. It is clear that TFOs 1-4 are bound within the crystals at both pH values. The scale bar represents 50 μm.

References

[1] M. J. Blommers, F. Natt, W. Jahnke, B. Cuenoud, *Biochemistry* **1998,** *37*, 17714–25.

[2] A. J. Hampshire, D. A. Rusling, V. J. Broughton-Head, K. R. Fox, *Methods* **2007**, *42*, 128–40.

[3] R. A. J. Darby, M. Sollogoub, C. McKeen, L. Brown, A. Risitano, N. Brown, C. Barton, T. Brown, K. R. Fox, *Nucl. Acids Res.* **2002**, *30*, e39.

[4] J. Zheng, J. J. Birktoft, Y. Chen, T. Wang, R. Sha, P. E. Constantinou, S. L. Ginell, C. Mao, N. C. Seeman, *Nature* **2009**, *461*, 74–77.
